# Management and outcome of obstructive airway complications after lung transplantation – a 12-year retrospective cohort study

**DOI:** 10.1177/17534666231181541

**Published:** 2023-08-01

**Authors:** Jens Gottlieb, Thomas Fuehner, Patrick Zardo

**Affiliations:** Department of Respiratory Medicine and Infectious Diseases OE 6870, Hannover Medical School (Medizinische Hochschule Hannover, MHH), Carl Neuberg Strasse 1, 30625 Hannover, Germany. German Center for Lung Research (DZL), Gießen, Germany; Department of Respiratory Medicine, Siloah Hospital, Hannover, Germany; Department of Cardiothoracic, Transplantation and Vascular Surgery, Hannover Medical School, Hannover, Germany

**Keywords:** biodegradable implants, bronchoscopy, graft survival, lung transplantation, stents

## Abstract

**Background:**

Obstructive airway complications (OACs) represent a significant problem after lung transplantation (LTx). Bilateral OACs after double lung transplantation are infrequently reported.

**Objectives:**

The aim of this study was to investigate management and outcome of OAC.

**Design:**

Retrospective single-center cohort study

**Methods:**

Adult patients with bilateral LTx performed between 2010 and 2021 were included. Patients with follow-ups of less than 3 months and after heart–lung transplantation were excluded. OAC was defined either as the need for stenting, surgical revision, or balloon dilatation. Outcome parameters included graft survival, graft function, quality of life, and management.

**Results:**

During the study period, 1,170 patients were included. Hundred thirty-five (11.5%) patients developed OAC. Forty-six (4.4%) patients had significant bilateral OAC. Thirty-seven (80%) bilateral OAC patients were treated by stent insertion; in 34 patients, biodegradable stents were used. The median number of bronchoscopies in bilateral OAC was 26 during the first postoperative year compared with nine in controls (*p* < 0.001). Fourteen OAC patients (*n* = 10 bilateral) underwent surgical revision including six re-do transplantations. Graft loss occurred significantly more frequently in patients with bilateral OAC with a graft survival of 63% and 50% in these after 3 and 5 years compared with 83% and 73% in controls without OAC (*p* < 0.001). Baseline forced expiratory volume in 1 s (FEV1) in patients with bilateral OAC was median 58% predicted in comparison with 90% in controls (*p* < 0.001). Quality of life was significantly reduced.

**Conclusion:**

Bilateral OACs impose a high burden of disease on patients after lung transplantation and were associated with early and late graft loss. Affected patients’ OAC demonstrated reduced graft function and impaired quality of life. Most OACs were managed by bronchoscopy preferably by non-permanent stenting. Surgery including re-do transplantation was used in selected cases.

## Introduction

Airway complications represent a significant problem after lung transplantation (LTx). Airway complications can be divided into obstructive and necrotic complications. The latter occur within weeks after LTx and are nowadays usually managed by observation only, but frequently result in later obstructive complications by aberrant wound healing. Obstructive airway complications (OACs) are more frequent and occur later than necrotic complications, usually within months after transplant. The onset of virtually all OAC is within the first postoperative year, and its reported incidence ranges from 2.6% to 24.6%.^1–10^ The proportion of bilateral OAC in patients after sequential bilateral LTx is seldom reported.

Pathogenesis and risk factors of airway complications after LTx have been studied in various publications.^5,11–13^ Extensive necrosis as a consequence of airway ischemia is the most important risk factor of later OAC. Fungal infections and surgical techniques are also associated with the development of airway complications but may be related to airway ischemia themselves. Airway ischemia in the post-anastomotic region is caused by several factors. During transplantation, the arterial systemic blood supply is not restored routinely. Spontaneous reestablishment of bronchial arterial circulation can take several weeks. The viability of the donor bronchus during this interval depends on the retrograde blood flow from the pulmonary circulation through collaterals. The surgical technique seems to play a role^14^ as well as postoperative management. Postoperative hypotension and volume depletion should be avoided.^12^ OAC may manifest clinically as malacia, strictures, or excessive granulation tissue, and management is usually done by bronchoscopy. Various endoscopic techniques are reported, including mechanical^15^ and thermal debulking (cryotherapy,^16^ laser,^17^ electrocautery,^18^ and argon therapy),^19^ balloon bronchoplasty,^20^ insertion of various types of stents,^3,21,22^ topical administration of drugs,^23^ and brachytherapy.^24^ Occasionally, surgery is performed in selected cases and OAC may be an indication for re-do transplantation.

OACs demonstrate a high rate of recurrence and may impair quality of life (QoL) due to frequent bronchoscopies, reduced graft function, and infectious complications, but the exact extent is unknown.

The aim of this study was to investigate the impact, management, and outcome of bilateral OACs in a large LTx cohort with a hypothesis of impaired graft survival and increased morbidity.

## Methods

A retrospective case–cohort study was performed in a large lung transplant program. The study was conducted following the ethical guidelines of the 1975 Declaration of Helsinki. Patients signed informed consent for anonymized data analysis in retrospective studies within the German Center of lung research. The use of data to conduct retrospective analysis was covered by the ethics committee’s vote (No 2923-2015, update September 24, 2021).

All adult patients with bilateral LTx (including combined non-thoracic and bilateral lung) performed between January 1, 2010 and December 31, 2021 were included. Patients with follow-up of less than 3 months were excluded as well as heart–lung transplant recipients because of a single tracheal anastomosis. For all patients, the first transplantation during the study period was included. Graft loss was defined as death or re-do transplantation.

Antifungal prophylaxis was applied to all patients for at least 6 months with azoles and additional inhaled liposomal amphotericin B for 3 weeks. Routinely, patients received a 1- to 2-week course of broad-spectrum antibiotics tailored to results of intraoperative cultures after surgery. In cases of suspected mucosal infection of the anastomotic and post-anastomotic regions, multiple biopsies were taken from these regions for cultures and histopathology. Fungal infection of the anastomotic and post-anastomotic regions was defined by macroscopic appearance plus positive cultures and/or the presence of fungal infection on histopathology.

Data were extracted from a database containing all transplant bronchoscopies (*n* = 32,920) performed by the Department of Respiratory Medicine since 2008. Additional clinical data were extracted from medical records. Follow-up ended on July 31, 2022 or at graft loss, whichever occurred first. Relevant OACs were defined as the need for stenting, surgical revision, or balloon dilatation (or mechanical dilatation by endoscopes larger than 6 mm outer diameter) as suggested in the literature.^7^ Airway complications were graded according to the S (stenosis) subdomain of the 2019 International Society of Heart and Lung Transplantation (ISHLT) consensus document,^25^ whenever photographs or videos of the anastomosis were available. Strategies to control observation were addressed by crosschecking selection by two investigators independently, and corroboration of multiple information sources (databases for bronchoscopy and transplantation plus medical records).

Lung-transplanted patients were followed in a specialized outpatient clinic in intervals depending on the time after transplantation and clinical stability. Loss of follow-up in our program is less than 5%. Bronchoscopy with broncho-alveolar lavage and transbronchial biopsy was performed routinely during the first year weekly until week 3 and the months 1, 3, 6, and 12 and whenever clinically indicated thereafter. Acute rejection was graded by histopathology according to current standards.^26^ In patients with an intervention for OAC, intervals between 1 and 4 weeks were used until clinical stability was reached. Case management by telephone or video consultations was offered to all patients between visits. At each appointment in the outpatient clinic history, physical exam, spirometry, and laboratory tests including immunosuppressant levels were obtained. Self-rated levels of health perception and QoL were assessed routinely on each visit using a visual analog scale by asking patients: ‘On a scale of 0 to 10, with 10 meaning perfectly healthy, what is you perceived health today?’.^27^

All endoscopic interventions for OAC were performed by experienced pulmonologists. The majority of interventions were performed with flexible bronchoscopes (BF-1TQ180, BF1T180, BF-1TH190, BF- P190, BF 3C40, all Olympus, Tokyo, Japan). Endoscopic treatment was based on underlying problems (e.g. granulation tissue, malacia, strictures) and time after transplant. In brief, early after LTx (first 3 months), mechanical debridement by forceps was undertaken and after 3 months excessive granulation tissue leading to airway obstruction was removed by thermal therapy (argon plasma, coagulation, electrocautery, or cryotherapy, Erbe, Tübingen, Germany). Strictures were treated by balloon dilatation (CRE^™^ graduated Pulmonary Balloon Dilatation Catheter 6–8 or 8–10 mm, Marlborough, MA, United States). Recurrent strictures after more than two dilatation procedures or significant malacia were treated with biodegradable (DV stent, ELLA_CS, Hradec Králové Czech Republic) or self-expandable metal stents (SEMS) (Bronchus Stent TTS, Alveonova, Aachen, Germany) at least 6 months after surgery. Stents were inserted in general anesthesia and stents were deployed via direct vision as previously described.^22^ Routine endoscopic follow-up after stent insertion was performed after 3 days, and then in 2- to 4-week intervals until 4 months. Topical mitomycin or paclitaxel drug-coated balloons were used in recurrent strictures occasionally until 2015. Residual strictures were defined as freedom from any intervention for at least 3 months on the last follow-up and a reduction of more than 50% in the cross-sectional area on a lobar level on bronchoscopy.

Transplant procedures were performed by cardiac surgeons. Surgical revisions for OAC were performed by thoracic surgeons.

Spirometry was performed according to American Thoracic Society/European Respiratory Society guidelines^28^ with reference values of forced expiratory volume in 1 s (FEV1) and forced vital capacity (FVC) based on European Community for Steel and Coal.^29^ Chronic lung allograft dysfunction (CLAD) was defined as persistent FEV1 < 80% in of the baseline FEV1 according to recently established criteria.^30^

Baseline allograft dysfunction was defined by FEV1 and FVC below 80% predicted as proposed by Liu *et al*.^31^

### Statistical analysis

Statistical analysis was performed with metric variables expressed as medians, and 25% and 75% quartiles, and categorical variables by absolute numbers and percentage of data entries. Univariate analyses were performed using the median test for continuous variables and the chi-square test or Fisher’s exact test for categorical variables. Survival analysis was performed using the Kaplan–Meier method. The last observation was the last visit of the patient. Cox regression multivariate analysis was conducted to analyze survival. The level of significance was set at ⩽ 0.10 for including variables identified by univariate analysis between groups. Two control groups for bilateral OAC were used (1) patients without OAC and (2) patients with unilateral OAC to study the effect magnitude. No imputation for missing data was performed but variables with a proportion of > 30% missing were excluded.

## Results

**Figure 1.**
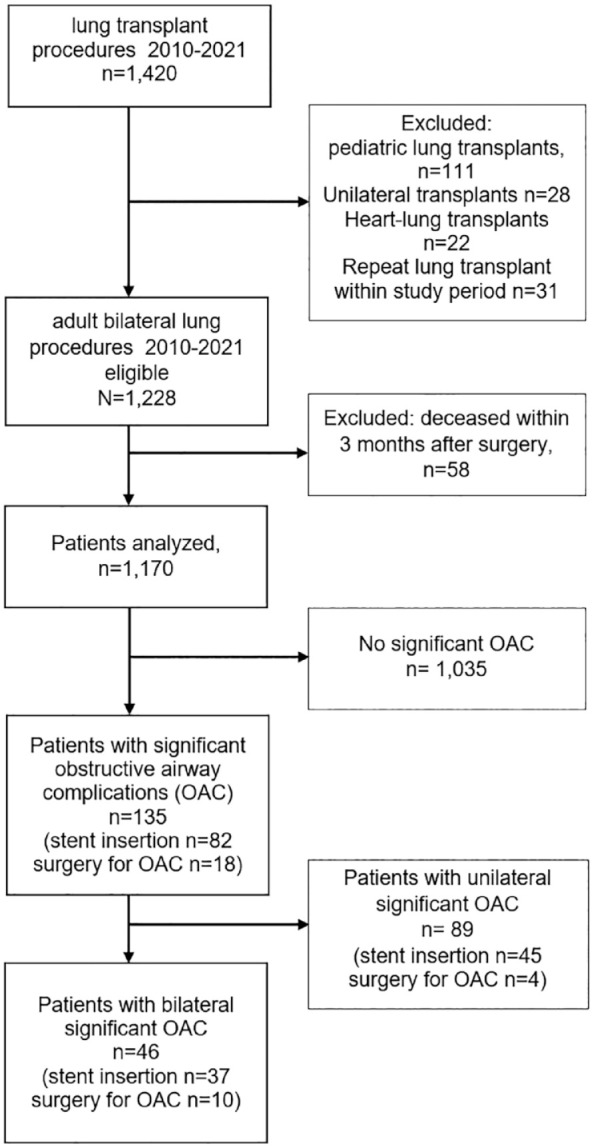
Flowchart of patients. OAC, obstructive airway complications.

Among 1,420 patients transplanted during the study period, 1,170 were included in our analysis (Figure 1). Hundred thirty-five (11.5%) patients developed a relevant OAC with a median onset of 84 days (25 and 75 quartile; 63, 112 days) after LTx. Forty-six (4.4% of the total cohort and 34% of all OACs) patients had significant bilateral OAC. The incidence of bilateral OAC increased from 3.1% (26 cases of 835 LTx) between 2010 and 2018 to 10.0% (20 of 200) from 2019 to 2021. The proportion of bilateral OAC of all OACs reached 53% in the most recent period.

Patient demographics are displayed in Table 1. Lung–liver transplantation was the underlying procedure in five patients, two of these experienced significant unilateral OAC and no lung–liver recipient developed bilateral OACs.

**Table 1.** Patient characteristics.

Variable	Patients without OAC (*n* = 1,035)	Patients with unilateral OAC (*n* = 89)	Patients with bilateral OAC (*n* = 46)	*p*-value
Sex, *n* (%)				
Female	499 (48)	30 (34)	12 (26)	< 0.001
Male	536 (52)	59 (66)	34 (74)	
Age at transplant, median years (25th, 75th percentiles)	43 (53, 59)	40 (51, 57)	40 (54, 59)	0.169
Recipient smoking history, *n* (%)	516 (50)	36 (40)	29 (64)	0.041
Steroid use pre-transplant, *n* (%)	393 (37)	30 (35)	15 (32)	0.650
Body mass index, median kg/m^2^ (25th, 75th percentiles)	21 (19, 24)	21 (19, 23)	22 (18, 23)	0.311
Lung allocation score^a^ at transplant, median (25th, 75th percentiles)	36 (33, 42)	34 (32, 38)	37 (32, 41)	0.112
Bridged by mechanical support,^b^ *n* (%)	74 (7)	6 (7)	1 (2)	0.431
Diagnosis, *n* (%)				
Emphysema/alpha-1 antitrypsin deficiency	324 (31)	27 (30)	10 (23)	0.224
Fibrosis/interstitial lung disease	331 (32)	28 (31)	14 (32)	
Cystic fibrosis/bronchiectasis	177 (17)	22 (24)	13 (30)	
Pulmonary hypertension/vascular diseases	57 (6)	1 (1)	1 (2)	
Other	146 (14)	13 (14)	6 (14)	

OAC, obstructive airway complication.

a Available in 985, 74, and 41 patients.

b Pre-transplant extracorporeal support or invasive mechanical ventilation.

In patients with available photo or video documentation (*n* = 122), six (5%) had isolated anastomotic lesions, 92 (75%) had anastomotic plus lobar/segmental lesions, and 24 (20%) had lobar/segmental lesions only according to the ISHLT consensus document. All OAC lesions had a more than 50% reduction in cross-sectional area (extent c and d).

### Endoscopic management

In 271 (23%) patients, endoscopic recanalization for excessive granulation tissue was performed on at least one occasion early after transplantation. The first endoscopic recanalization occurred after a median of 84 days (25% and 75% quartiles, 63, 112 days). Fifty percent of patients with early endoscopic recanalization developed later significant OAC. Eighty-two (61%) patients were treated by stent insertion (Table 2) and 135 (11.5%) patients by endoscopic dilatation. First dilatation was performed median of 172 days after transplantation (25% and 75% quartiles, 111 and 215 days). Balloon dilatations were repeatedly performed in 73% of patients with a median of two sessions (25% and 75% quartiles, 1 and 4). Most patients (86%) were treated with biodegradable stents, in nine patients, both SEMS and biodegradable stents were used. Slightly more target lesions in significant OAC (*n* = 98, 54%) were located on the right side than on the left side (*n* = 83, 46%). The proportion of patients receiving stents increased from 7.1% (59 of 835) from 2010 to 2018 to 11.5% (23 of 200) between 2019 and 2021.

**Table 2.** Treatment and outcomes.

Variable	Patients without OAC (*n* = 1,035)	Patients with unilateral OAC (*n* = 89)	Patients with bilateral OAC (*n* = 46)	*p*-value
Graft loss, *n* (%)	340 (33)	24 (27)	24 (52)	0.011
Death, *n* (%)	316 (31)	20 (23)	15 (33)	0.261
Re-do transplantation, *n* (%)	24 (2)	4 (4)	9 (20)	0.019
Days until first outpatient visit, median days (25th, 75th percentiles)	27 (31, 39)	26 (31, 35)	27 (32, 45)	0.417
Bronchoscopies during first postoperative year, median (25th, 75th percentiles)	9 (8, 11)	21 (17, 27)	26 (31, 40)	< 0.001
Anastomotic dehiscence, *n* (%)	22 (2)	11 (13)	11 (24)	< 0.001
Number of transbronchial biopsies within first 2 postoperative years, *n*	4.495	907	94	
Biopsy grade A1, *n* (%)	529 (12)	125 (14)	12 (13)	0.226
Biopsy grade A2 and above, *n* (%)	160 (3)	29 (3)	2 (2)	0.701
Biopsy proven bronchial fungal infection within first 6 postoperative months, *n* (%)	37 (4)	5 (6)	3 (7)	0.396
Stents per patient, median (25th, 75th percentiles)	–	1 (0, 1)	1 (1, 2)	< 0.001
Use of biodegradable stents, *n* (%)	–	44 (48)	34 (77)	0.001
Use of self-expandable metal stents, *n* (%)	–	7 (8)	6 (14)	0.272
Use of topical mitomycin or paclitaxel, *n* (%)	–	9 (10)	1 (2)	0.095
Airway colonization, *n* (%)	120 (12)	8 (9)	16 (35)	< 0.001
Chronic lung allograft dysfunction, *n* (%)	289 (28)	22 (24)	6 (14)	0.117
Baseline FEV1 < 80% predicted,^a^ *n* (%)	299 (30)	47 (57)	33 (79)	< 0.001
Baseline FVC < 80% predicted, a *n* (%)	170 (18)	18 (22)	18 (43)	< 0.001
Baseline FEV1 and FVC < 80% predicted,^a^ *n* (%)	61 (7)	13 (17)	8 (25)	< 0.001

FEV1, forced expiratory volume in 1 s; FVC, forced vital capacity; OAC, obstructive airway complication.

a *n* = 914, *n* = 80, and *n* = 30 patients evaluable.

Hundred fourteen biodegradable stents were inserted in 78 patients during the study period. Twenty-two patients received more than one biodegradable stent. Early migration leading to stent extraction occurred in two stent procedures (2%) and two patients. Copious secretions with the need for bronchoscopic intervention were observed in 10 cases (9%). Granulation tissue with stent obstruction leading to endoscopic recanalization occurred in 15 (13%) cases. Re-stenosis after stent degradation was observed in 21 cases (18%). No death related to the insertion of a biodegradable stent was noted.

First-year bronchoscopies (Table 2) were increased by 178% in patients with OAC compared with those in patients without OAC. Stents were used more frequently in bilateral OAC than in unilateral OAC. Biodegradable stents were used most frequently.

Significantly, more endoscopic interventions were performed in patients with OAC during the first postoperative year (Table 2). Freedom from any intervention was 82%, 93%, and 98% after 1, 2, and 3 years, respectively, following transplantation in patients with OAC. In 84 (62%) OAC patients, residual strictures were present during the latest follow-up bronchoscopy. In 72 (86%) of these patients, the middle lobe was involved, in 40 (48%) patients, upper lobes were affected, and in 9 (11%) patients, lower lobes could not be intubated. In 36 (43%) patients, more than one residual stricture on the lobar level was present. Residual strictures were more frequent in recipients affected by bilateral OAC (*p* < 0.001). Thirty-eight percent of these patients had at least one lobar bronchus occluded or severely narrowed. In 22 (48%) patients, more than one lobe was involved. In unilateral OAC patients, 53% had at least one residual stricture, and in 16%, more than one lobe was affected.

### Thoracic surgery

Fourteen patients underwent thoracic surgery for OAC including six re-do transplantations with OAC (all were bilateral OAC) as the primary indication (*n* = 5 bilateral, *n* = 1 unilateral). Sleeve resection was performed in four patients (three with bilateral OAC), and two cases of lobectomy and each a single case of bi-lobectomy (all lobectomies were in unilateral OAC patients) and pneumonectomy (a patient with bilateral OAC) due to infectious post-obstructive complications. No recurrence of significant OAC was observed after thoracic surgery for OAC. Re-do transplantations were performed after a median of 217 days after primary transplantation. All patients undergoing thoracic surgery for OAC underwent prior balloon angioplasty and nine had prior stent insertion. Three patients with bilateral OAC underwent later re-do transplantation due to CLAD.

### Graft survival

**Figure 2.**
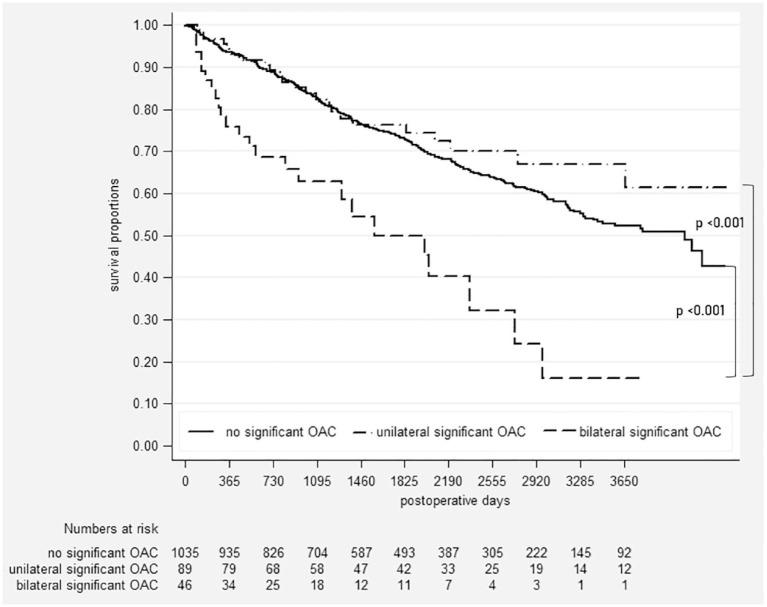
Kaplan–Meier curve of graft survival. OAC, obstructive airway complications.

Three hundred fifty-one patients died during follow-up, 20 deaths occurred in patients with unilateral OAC, and 15 died in the subgroup of patients with bilateral OAC. Thirty-seven patients underwent re-do transplantation, four in the subgroup with unilateral OAC, and 15 (33%) in the subgroup with bilateral OAC. The majority of graft losses were attributed to death in all groups and the proportion of patients undergoing re-do transplantation increased from 2% in patients without OAC to 4% and 20% in patients with unilateral and bilateral OACs, respectively (Table 2). Overall graft loss occurred significantly more frequently in the subgroup of patients with bilateral OAC (Table 2 and Figure 2). Graft survival after 1, 3, and 5 years was 93/83/73%, 93/83/75%, and 76/63/50% in patients without, with unilateral, and with bilateral OACs, respectively (Figure 2). There was no difference in survival between patients with unilateral OAC and patients without OAC (log-rank *p* = 0.279). Development of bilateral OAC was independently associated with graft loss with an adjusted hazard ratio of 2.31 (95% confidence interval: 1.27, 4.18; see Table 3). Causes of death (*n* = 15) in patients with bilateral OAC were respiratory failure in seven, infection/sepsis in four, pulmonary bleeding in one, malignancy in one, and CLAD in two.

**Table 3.** Survival analysis.

Covariate	*N*	Survival (months), median (95% CI)	Univariate		Multivariate	
			Hazard ratio (95% CI)	*p*	Hazard ratio (95% CI)	*p*
Age group, *n* (%)						
18–46 years	359	66 (58, 71)	(Ref.)	(Ref.)	(Ref.)	(Ref.)
47–53 years	239	62 (52, 67)	**1.33 (1.00, 1.764)**	**0.047**	1.21 (0.83, 1.76)	0.327
54–59 years	345	49 (42, 58)	1.26 (0.96, 1.65)	0.09	1.26 (0.85, 1.87)	0.253
60 years and older	227	46 (40, 56)	**1.61 (1.22, 2.14**	**0.001**	1.47 (0.98, 2.19)	0.061
Sex, *n* (%)						
Male	628	57 (51, 61)	(Ref.)	(Ref.)		
Female	539	58 (50, 62)	1.10 (0.03, 1.35)	0.335		
Primary disease, *n* (%)						
Emphysema incl. alpha-1 antitrypsin deficiency	361	49 (44, 59)	(Ref.)	(Ref.)	(Ref.)	(Ref.)
Pulmonary vascular disease	59	52 (42, 69)	0.92 (0.57, 1.475)	0.723	1.13 (0.66, 1.942)	0.656
Cystic fibrosis/bronchiectasis	212	67 (62, 74)	**0.61 (0.44, 0.85)**	**0.003**	0.81 (0.51, 1.29)	0.379
Fibrosis/interstitial lung disease	373	55 (47, 60)	1.05 (0.82, 1.35)	0.696	1.02 (0.77, 1.35)	0.888
Other (incl. re-do transplantation)	165	55 (41, 64)	1.33 (0.99, 1.79)	0.06	1.09 (0.74, 1.59)	0.669
Chronic lung allograft dysfunction, *n* (%)						
No	828	62 (54, 69)	(Ref.)	(Ref.)	(Ref.)	(Ref.)
Yes	317	57 (51, 60)	2.37 (1.93, 2.91)	0.422	**2.62 (2.09, 3.29)**	**< 0.001**
Metastatic tumor disease after transplantation, *n* (%)						
No	1,124	58 (52, 61)	(Ref.)	(Ref.)	(Ref.)	(Ref.)
Yes	46	41 (31, 64)	3.87 (2.83, 5.28)	< 0.001	**3.80 (2.69, 5.39)**	**< 0.001**
Obstructive airway complication, *n* (%)						
No	1,035	58 (53, 61)	(Ref.)	(Ref.)	(Ref.)	(Ref.)
Unilateral	89	55 (42, 70)	0.82 (0.55, 1.23)	0.336	0.75 (0.46, 1.22)	0.241
Bilateral	46	31.5 (19, 43)	**2.40 (1.57, 3.66)**	**< 0.001**	**2.31 (1.27, 4.18)**	**0.006**
Baseline lung allograft dysfunction *n* (%)						
No	942	61 (58, 65)	(Ref.)	(Ref.)	(Ref.)	(Ref.)
Yes	82	55 (44, 68)	1.43 (0.99, 2.07)	0.055	1.41 (0.96, 2.08)	0.083

CI, confidence interval. Bold values were associated with mortality.

### Graft function

**Figure 3.**
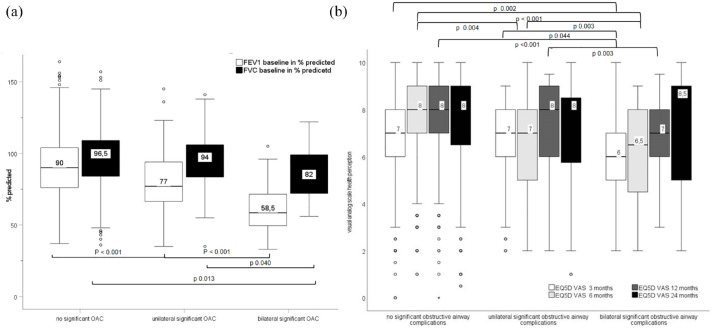
Baseline graft function (a) in percent predicted and patient reported health perception (b, visual analogue scale). EQ5D, patient reported EuroQol health perception questionnaire; FEV1, forced expiratory volume in 1 s; FVC, forced vital capacity, OAC, obstructive airway complications; VAS, visual analogue scale.

Baseline FEV1 was significantly lower in patients with OAC in comparison with patients without OAC (Figure 3(a)) and effects were more pronounced in patients with bilateral OAC resulting in a median baseline FEV1 of 58% predicted in comparison with 90% predicted in recipients without OAC. In contrast to patients affected by unilateral OAC only, bilateral OAC patients had an additional and significant reduction of FVC to a median 82% compared with 97% in patients without OAC. Baseline allograft dysfunction was present in every fourth recipient affected by bilateral OAC and this was significantly increased in comparison with controls (Table 2).

Three hundred seventeen patients developed CLAD; 289 (28%) patients without OAC developed CLAD, 22 (25%) patients with unilateral OAC, and six CLAD patients had bilateral OAC. The median time to development of CLAD was shorter in patients with bilateral OAC (545 days) in comparison with patients with unilateral (717 days) or without OAC without reaching statistical significance (*p* = 0.142).

### Quality of life

Patient’s health perception on a visual analog scale was significantly reduced in patients with bilateral OAC up to 1 year after LTx. Within the first 6 months, also patients with single-sided OAC reported a reduced health perception compared with controls without OAC (Figure 3(b)).

## Discussion

This study demonstrates that bilateral OACs have a considerable negative impact on graft survival, graft function, long-term complications, and early QoL in LTx patients. Endoscopic management of bilateral OAC is challenging and most patients are treated by stent insertion, some will benefit from surgical revision.

Published data reporting the incidence of bilateral OAC after LTx are scarce. Bilateral OAC affected just a single LTx patient of 1,555 (0.1%) in a single-center study^7^ and less than 2% of all anastomotic airway complications were bilateral in another single-center analysis of 490 patients after transplantation.^10^ When applying the same definition of OAC as the Vienna group, our overall incidence was more than four times higher and the proportion of bilateral OAC was almost 40-fold increased in our program. Risk factor analysis is beyond the scope of our article, but surgical technique and early postoperative management to optimize perfusion seem to be surgical for prevention of later OAC. Airway ischemia seems to be the most important risk factor for OAC.^12^ A lower incidence of OAC has been reported uniformly by centers in which only thoracic surgeons perform airway anastomosis in LTx.^7,10,13,14^

There are conflicting reports about the impact of OAC on survival in lung transplant recipients. Murthy *et al.*^5^ reported an impaired 2-year survival of 60% in 67 patients affected by OAC in comparison with 75% of controls. Also, a registry analysis of 233 cases reported a 3-year survival of 39% compared with 68% in controls.^32^ In 111 LTx patients treated with SEMS after LTx, 5-year survival was 60% compared with 76% in controls.^33^ No difference in survival was reported by two other authors^4,6^ involving 50 recipients in total. In our study, a negative impact on survival was only observed in transplanted patients with significant bilateral OAC.

To our knowledge, there are few publications focusing on the effect of OAC on baseline FEV1. Mazzetta *et al.*^4^ reported a 25% lower baseline FEV1 in 22 bilateral LTx patients affected by OAC compared with 139 controls without differentiating between unilateral or bilateral OAC. In another retrospective study, 6-month FEV1 in percent predicted was reduced from 68 to 58.5 (relative –17%) in 34 patients with OAC.^2^ In our study, patients with bilateral OAC lost 35% of baseline FEV1, which confirms this observation. Lower baseline FEV1 is associated with impaired survival in various publications^31,34,35^ and was associated with a higher incidence of CLAD or earlier onset.^34–36^ Baseline allograft dysfunction may explain the earlier onset of CLAD in our cohort, although this missed statistical significance is probably explained by low patient numbers.

In multivariate analysis, bilateral OAC but not BLAD was associated with graft loss in our study. Retrospective studies from Strasbourg^4^ and Temple University reported^1^ increased infection rates in 35 and 22 patients affected by OACs, respectively. Our group published in 2010 a 45% rate of airway colonization in patients treated with SEMS.^3^ No details were available from the two other publications about their use of stents and airway colonization. Because of the predominant use of biodegradable stents in 82 patients of our study, the use of stents alone might not explain an increased risk of colonization in OAC patients. A median time to stent degradation of 4.5 months is observed in LTx patients and significant improvement of pulmonary function after insertion.^22,33^ Duration of follow-up bronchoscopies decreased after stent insertion in most of our patients and the majority (app. 60%) received bronchoscopy just for airway clearance. Nevertheless, residual strictures causing impairment in lung function and increased rate of infection were frequent in our cohort even when target lesions remained patent after degradation of stents. The idea of non-permanent stenting in anastomotic complications after LTxs and in benign airway stenosis, in general, is attractive and can be achieved by silicone stents as well.^21^ In 17 patients treated with silicone stents after LTx, airway colonization was noted in all patients.^11^ Airway colonization especially with pseudomonads was associated with an increased risk of CLAD in several publications.^37–39^ In our view, silicon stents are less suitable for LTx patients due to potential kinking in complex lesions, no collateral ventilation without customizing, reduced inner diameter, and high risk of colonization.

To our knowledge, there is no published data on the QoL in patients with OAC after LTx. The group from Strasbourg reported similar dyspnea grades in 22 OAC patients.^4^ Airflow limitation might not be the only compound of health perception given the near only slightly reduced baseline FEV1 below the lower limit of normal in unilateral OAC patients. The burden of frequent bronchoscopies and emergency visits may represent an additional factor in reduced QoL within the first postoperative year.

The additional use of health care resources is reflected by rocketing first-year bronchoscopies in patients with bilateral OAC. A 27-year-old female cystic fibrosis patient with bilateral OAC underwent 68 bronchoscopies – most of them with interventions – within the first 11 months after LTx until finally undergoing successful re-do transplantation. The mean cost based on capital investments, repairs, and reprocessing costs of reusable bronchoscopes was calculated to be US$266.^40^ In our program, 2,160 additional bronchoscopies during the 12-year period for endoscopic management of OAC patients were needed. With conservatively estimated costs of 600 Euros per bronchoscopy, including additional material for interventions (e.g. balloons) and staffing, this resulted in additional costs of almost 1.3 million Euros during that period.

The limitation of our study is a single-center design. Management of OAC is highly individualized between centers and the incidence is highly variable. The definition of OAC may vary between centers although we decided to use the pragmatic definition used in a recent publication by a similar-sized program. The ISHLT consensus is difficult to apply retrospectively, although most of our interventions were videotaped. Also, the timing for balloon dilatation and stent insertion during the management and evolution of OAC may be different between different programs. Usually, surgery for OAC is regarded as a last resort in management and it is unknown if earlier operative measures may result in better outcomes. As in other orphan procedures, there is a lack of randomized controlled trials in management.

## Conclusion

Bilateral OACs impose a high burden of disease on patients after LTx. Bilateral OAC was associated with early and late graft loss, and graft colonization. Patients with bilateral OAC demonstrated significantly reduced graft function and affected patients reported a significantly impaired QoL. These complications were mostly managed endoscopically, preferably by non-permanent stenting in our study with high utilization of health care resources. Prevention of OACs is crucial.

## Declarations

**Ethics approval and consent to participate:** The authors are accountable for all aspects of the work in ensuring that questions related to the accuracy or integrity of any part of the work are appropriately investigated and resolved. The study was conducted in accordance with the Declaration of Helsinki (as revised in 2013), available at: https://www.wma.net/wp-content/uploads/2016/11/DoH-Oct2013-JAMA.pdf (as revised in 2013). The study was approved by institutional ethics/committee of Hannover Medical School (No 2923-2015, update September 24, 2021) and informed consent was taken from patients.

**Consent for publication:** Not applicable, no individual patient data.

**Availability of data and materials:** Anonymized participant data will be made available after publication upon requests directed to the corresponding author. Proposals will be reviewed and approved by the investigators and collaborators on the basis of scientific merit.
